# The role of perceived risk on dishonest decision making during a pandemic

**DOI:** 10.1111/risa.14082

**Published:** 2022-12-12

**Authors:** Ian G. J. Dawson, Yaniv M. Hanoch

**Affiliations:** ^1^ Centre for Risk Research, Southampton Business School, Highfield Campus University Of Southampton Southampton UK

**Keywords:** COVID‐19 pandemic, decision making, dishonesty, risk perception

## Abstract

The COVID‐19 pandemic presented serious risks to the health and financial wellbeing of millions of people across the world. While many individuals adapted to these challenges through a variety of prosocial and protective behaviors (e.g., social distancing, working from home), many others also engaged in dishonest behaviors (e.g., lying to obtain vaccines or furlough payments). Hence, the COVID‐19 pandemic provided a unique context in which to obtain a better understanding of the relationship between risk and dishonesty. Across three preregistered studies, we assessed whether objective risk and perceived risk influenced the decision to behave dishonestly in order to gain access to vaccines and furlough payments during a pandemic. We also assessed the extent to which such dishonesty was deterred by the probability of the dishonesty being detected. We found that heightened health risk perceptions were positively related with lying to obtain a vaccine (Studies 1 and 2), but found no evidence of the same relationship between financial risk perceptions and lying to access furlough payments (Study 2). We also found that the probability of dishonesty being detected had a negative relationship with dishonest behavior (Study 3). In addition, across the three studies, we found that (i) dishonesty was consistently evident in approximately one‐third of all of our samples, and (ii) greater dishonesty was associated with older age. We discuss how our findings could be utilized by policy makers to better deter and detect dishonest behaviors during future similar crises.

## 1 INTRODUCTION

In March 2020, the World Health Organization (WHO) declared the rapid spread of the coronavirus disease (COVID‐19) as a global pandemic (Cucinotta & Vanelli, 2020). Subsequently, a wide range of protective measures (e.g., vaccines, furlough schemes) was implemented by governments across the world. While these measures were instigated to help people, communities, and organizations, they often inadvertently presented individuals with opportunities to gain personal advantages through dishonest behaviors. For example, evidence suggests that many individuals provided false information in order to obtain COVID‐19 vaccinations (Chen, 2021; UK Government, 2021a; Williams, 2021), and that this behavior was largely driven by a fear of the severe and potentially life‐threatening effects of the disease (e.g., Davies & Devlin, 2021; Hossesini, 2021). Similarly, other measures introduced during the COVID‐19 pandemic presented opportunities for financial fraud. In January 2022, the UK government wrote off over £4 billion in fraudulent COVID‐19 loans (Islam, 2022), and in the United States the number rose to an estimated $400 billion (Salmon, 2021). In most cases, individuals lied about their employment status or business practices to obtain furlough payments and other types of financial support. One reason for this magnitude of dishonesty during the pandemic could have rested with the lackluster enforcement mechanism that some governments employed and, thus, individuals soon became aware that the likelihood of their dishonesty being detected was negligible. Indeed, previous studies have found that a chief deterrent in reducing crime and dishonesty is a high probability that such behaviors will be detected (Kaushik et al., 2022; Nagin & Pogarsky, 2003; United States Department of Justice, 2016). Thus, a pandemic presents a unique context in which to examine whether an individual's willingness to lie is influenced by the magnitude of the risk faced (e.g., the lethality of the virus), by the nature of the risk faced (e.g., financial or health), and by the probability of being detected (e.g., 1 in 10 vs. 1 in 100). We present three studies that examine these three key issues.

### 1.1 Dishonesty and the health risks of COVID‐19

In December 2020, a 90 year‐old woman became the first person in the United Kingdom to receive a COVID‐19 vaccination outside of a clinical trial (BBC, 2020). In most countries, only a limited supply of COVID‐19 vaccines were initially available, which meant that governments had to prioritize who would receive the vaccine first (e.g., older people, individuals with specific health conditions). However, several reports have highlighted that fear of COVID‐19 may have motivated many individuals to lie in order to bypass vaccination criteria‐based selection processes (Chen, 2021; Williams, 2021). Indeed, a wealth of previous research shows that risk plays a central role in many decisions and behaviors, and that there is a strong positive relationship between perceived risk and the decision to engage in self‐protective behaviors (Dohle & Dawson, 2017; Floyd et al., 2000; Slovic, 1999; Weinstein et al., 1991). Notably, several studies indicate that the willingness to engage in health‐protective behaviors (e.g., vaccinations, self‐examinations) is often greatest among those with a heightened perceived risk of adverse health outcomes (Brewer et al., 2004, 2007; Weinstein et al., 1991).

Studies have also revealed that the perceived health risk of COVID‐19 has been a key driver of protective behaviors, such as wearing facemasks and social distancing (Garfin et al., 2021; Joslyn et al., 2021). However, it remains empirically untested as to whether such risk perceptions would be substantial enough to drive dishonest behaviors (e.g., lying about one's health status) in an effort to obtain a COVID‐19 vaccination. Given that risk‐taking behaviors can be motivated by desperation (Connors, 1992; Tucker & Ferson, 2008), it is possible that heightened risk perceptions might motivate dishonest behaviors that help to obtain a COVID‐19 vaccination. Hence, in the first of our three studies, we examined whether the magnitude of the health risk posed by a pandemic disease is positively related to lying in order to access a vaccination. Specifically, we tested whether people would lie to increase their chances of receiving a COVID vaccination and whether such dishonesty was positively related to the objective (i.e., communicated) and/or perceived risk of the disease. We assessed the influence of both objective and perceived risk because the two constructs can vary independently and, therefore, can have differing influences on decision‐making behaviors (Boholm, 1998; Hermansson, 2011; Knuth et al., 2013). For example, studies have found that objective health risk‐related information can be interpreted differently between individuals and that this can lead to variations in the willingness to engage in precautionary behaviors (see Reyna & Brainerd, 2007; Timmermans et al., 2008). In consideration of this literature, we hypothesized the following:

**H1**: Dishonesty increases as the objective health risk of a pandemic disease increases.
**H2**: Dishonesty increases as the perceived health risk of a pandemic disease increases.

### 1.2 Dishonesty and the financial risks of COVID‐19

Adverse health effects are, of course, only one consequence of COVID‐19. The pandemic has also had a substantial impact on the financial well‐being of millions of people. During the height of the pandemic, many countries across the globe initiated lockdowns and/or other restrictions on human interactions, which inflicted an enormous financial burden on many individuals and businesses. A study by Khetan et al. (2022), with over 24,000 participants from across the globe, reported that one‐third of the sample had experienced severe negative financial consequences, and close to 10% had lost their jobs. Data from the US labor market revealed that the unemployment rate in April 2020 reached over 14%, the highest rate since 1948 (US Congressional Research Service, 2021). Similarly, in the United Kingdom, close to 12 million jobs were furloughed (UK Parliament, 2021). Thus, for millions of individuals, job retention and financial support schemes represented their main or only source of income.

Research has shown that economic hardship is commonly associated with higher levels of dishonesty‐related crimes, such as fraud and tax evasion (Australian Institute of Criminology, 2021; Dang & Tran, 2021; United Nations Office on Drugs and Crime, 2012). Relatedly, during the COVID‐19 pandemic, the strict lockdowns conditions indirectly reduced the rate of location‐dependent crimes (e.g., burglary, assaults; Langton et al., 2021), but indirectly increased the rate of remotely committed crimes (e.g., fraud, identity theft; Deloitte, 2022; Price Waterhouse Cooper, 2022; UK Finance, 2022). For example, Google reported an unprecedented number of phishing emails during the pandemic (over 18 million per day), and the UK's Office for National Statistics (ONS) recorded an 85% increase in computer misuse crimes in 2021 (Interpol, 2020; ONS, 2021). Hence, it seems that during the pandemic, the subsequent economic downturn and the restricted capacity to commit location‐dependent crimes may have contributed to a marked rise in dishonest crimes and behaviors.

Interestingly, in their extensive review of the literature on dishonesty, Jacobsen et al. (2018), did not examine whether major crises (e.g., pandemics) influence dishonest behaviors, despite crises often creating opportunities to cheat (Federal Bureau of Investigation, 2022; Federal Communications Commission, 2022). Recent studies have found that during the COVID‐19 pandemic, many individuals engaged in dishonest behaviors, such as concealing COVID‐19 symptoms and disobeying lockdown/quarantine rules (Donnarumma & Pezzulo, 2021; O'Connor & Evans, 2020). However, these studies did not determine the extent to which the perceived financial risks imposed by the disease might motivate dishonest behaviors (e.g., lying about one's income) that could help to secure financial support. The possibility that financial risk perceptions and finance‐related dishonesty are positively related during a pandemic remains empirically untested. Yet, this relationship seems likely given that crimes involving dishonesty tend to increase when financial adversity looms. Hence, we hypothesized the following:

**H3**: When applying for furlough payments, dishonesty increases as the perceived risk of financial hardship increases.

Moreover, whether people are more or less likely to lie to protect their health or their financial position during a pandemic is also unknown. In light of the vast and well‐documented magnitude of the financial fraud that occurred in multiple countries during the COVID‐19 pandemic, we hypothesized the following:

**H4**: Dishonesty is greater when attempting to obtain furlough payments than when attempting to obtain COVID‐19 vaccinations.

### 1.3 COVID‐19, dishonesty, and the probability of detection

With dishonesty being so prolific and the consequences often being substantial and far reaching for society, academics have developed a keen interest in understanding dishonest behaviors (Gerlach et al., 2019). One explanation of dishonest behavior comes from economic theories, which assume that people weigh the benefits and costs of acting dishonestly (Allingham & Sandmo, 1972; Becker, 1968). That is, people first evaluate the expected benefit, the expected probability of being caught and the expected punishment. Then, by computing these three facets, they decide whether dishonesty will maximize their self‐interest. If it does, then it becomes a viable strategy. However, dishonesty is avoided when the probability of detection is perceived as too high and the punishment as too severe (see Hechter, 1990; Lewicki, 1984). This idea—also known as deterrence theory—is a pillar of economics and theories about crime and punishment (see Pratt et al., 2006). However, despite its appeal, there is limited empirical support for the notion that the punishment alone is an effective deterrent (Trang & Brendel, 2019). A report by the United States Department of Justice (USDJ) concluded that simply sending people to prison or “*increasing the severity of the punishment does little to deter crime*” (2016, p. 1). Rather, the USDJ argued that the key to deterring crime is by increasing the likelihood of being caught. This notion is supported by research evidence, which shows that dishonest behaviors are inversely related to the probability of detection (Kaushik et al., 2022; Nagin & Pogarsky, 2003). This suggests that a high likelihood of detection could play a key role in deterring dishonest behaviors during a pandemic.

High levels of fraudulent eligibility claims were made to financial support schemes during the first years of the COVID‐19 pandemic. Although some governments tried to deter such dishonesty by warning applicants that offenders would be prosecuted (UK Government, 2022; US Department of Justice, 2020), the high levels of fraudulent claims for financial support suggests that many people interpreted the probability of detection as being negligible. Hence, had there been a higher probability of dishonesty being detected, one might have expected to have observed less financial fraud during the pandemic. Furthermore, while it is plausible that the communicated probability (objective risk) and the subjective probability (perceived risk) of detection could both deter dishonesty during a pandemic, it is possible that one might be a greater deterent than the other. That is, the objective risk of detection (e.g., 20% detection rate) may act as an effective deterrent if brought to public attention. However, the objective probability could be open to subjective interpretation, whereby some individuals may form the subjective view that the objective probability represents a high risk of detection and others form the view that is represents a low risk of detection. Therefore, we considered that there would be value in testing the following two hypotheses:

**H5**: Dishonest behavior decreases when there is an increase in the objective risk of dishonesty being detected.
**H6**: Dishonest behavior decreases when there is an increase in the perceived risk of dishonesty being detected.

### 1.4 Research overview

We conducted three studies that were designed to respectively address three key knowledge gaps in the extant literature. Study 1 examined whether the magnitude of the health risk posed by a pandemic disease influenced the decision to lie to obtain a vaccine. We predicted that as the risk of the disease increased, so would the probability that individuals would lie in order to be vaccinated. Study 2 explored whether the propensity to lie was greater when attempting to obtain a vaccine or to obtain furlough payments. In light of the prolific financial fraud that occurred during the COVID‐19 pandemic, we predicted that lying would be more prevalent when attempting to obtain furlough payments. In Study 3, we manipulated the probability that lying would be detected, and predicted that as the risk of detection increased, lying would decrease.

## 2 METHODS AND RESULTS

### 2.1 Study 1

In this preregistered study (OSF preregistration: https://archive.org/details/osf‐registrations‐zvsju‐v1), we assessed the extent to which the risk of a highly infectious pandemic disease influences individuals to act dishonestly to protect themselves from that disease. We also assessed whether such dishonesty was positively related to the objective (i.e., communicated) and/or perceived risk of the disease. Hence, the purpose of Study 1 was to address Hypotheses 1 and 2.

#### 2.1.1 Participants

In September 2021, we recruited a sample of 305 adult participants via www.prolific.co and paid each participant £1.50. All participants were UK residents, with English as their first language and a prolific approval rating of ≥95%. Only participants who had received at least one COVID‐19 vaccination were recruited; this criterion was employed to ensure that any preexisting oppositional attitudes toward the vaccination would not bias responses to our stimulus materials. Although no participants failed our “instructional manipulation check” (IMC; Oppenheimer et al., 2009), three participants were excluded for providing incomplete data. This left a final sample of 302 participants. The sample's mean age was 31.9 (*SD* = 8.5), 265 identified as female and 141 had obtained a bachelor's degree or a higher qualification.

#### 2.1.2 Materials and procedure

Via a Qualtrics survey, participants responded to a purpose‐made scenario (see Supporting Information). The scenario asked the participants to imagine that, “*You are 40 years old, you have no known designated health conditions, and you have not experienced any flu‐like symptoms in the past year*.” It then explained that a deadly and highly contagious variant of COVID‐19, named “*COVID‐21,″*” was causing a global pandemic and that the participant could attempt to obtain a vaccination to protect themselves against the disease. To obtain the vaccine, participants would have to complete the national government's “*COVID‐21 Vaccine Registration Form*,” which asked participants 12 questions about their age and health status as described in the scenario. Participants were made aware that the government would prioritize giving the limited number of available vaccines (i.e., vaccines available for one in every 10 people) to individuals who met any of the 12 criteria respectively set out in each question. For example, one question in the form asked participants to state whether they had experienced flu‐like symptoms in the past 4 weeks and one question asked if they were 60 years or older. All 12 questions could be answers ‘yes’ or ‘no,’ with all ‘yes’ answers being deemed dishonest. All 12 questions were closely based on the 12 criteria that the UK's National Health Service (NHS) employed in 2021 to determine who should be prioritized for receiving the COVID‐19 vaccines (NHS, 2021). Using a between‐subjects design, the scenario was varied so that it described the objective risk of COVID‐21 as either two times, 10 times, or 100 times more deadly than all other known variants of COVID‐19. We elected to use three asymmetric (cf. equal) increases in objective risk because this enabled us to examine the influence a particularly wide range of objective risk levels across a small number of scenarios (for similar methodological approaches, see Baumgaertner et al., 2020; Dawson et al., 2012). Participants were randomly allocated to one of the three conditions.

To measure perceived risk, participants completed an 11‐point scale (0  =  not at all worried; 10  =  extremely worried) to indicate the extent of their worry about the potential effects of COVID‐21 on their health, and 101‐point scales (0  =  impossible; 100  =  certain) to indicate the likelihood that they would (i) become infected with COVID‐21, and (ii) experience life‐threatening symptoms if infected with COVID‐21. In addition, participants completed the 13‐item belief in a just world scale (Dalbert, 1999) and the 15‐item moral standards scale (Jones et al., 2000). These scales were used to help us explore, respectively, whether dishonesty might be influenced by the extent to which individuals believe that there is justice in the world and that their behavior is closely governed by an intrinsic set of moral principles. Finally, we used a single item to measure risk‐taking propensity (Dohmen et al., 2011), a 5‐point scale (1 = Left‐wing; 5 = Right‐wing) to measure political views, and an 11‐point scale (0 = Not at all willing; 10 = Extremely willing) to measure general willingness to receive vaccines.

#### 2.1.3 Analysis and results

To examine the responses to the 12 questions on the vaccine registration form, we created two versions of the dependent variable: (i) a binary variable that indicated whether the participant had or had not provided a least one dishonest answer, and (ii) a continuous variable consisting of the mean number of dishonest answers. We created these two conceptualizations of the dependent variable because the former measure (labeled ‘dishonesty binary’) showed if the participants had been dishonest, while the latter measure (labeled ‘dishonesty magnitude’) captured the *extent* to which the participants had been dishonest. Table 1 shows the counts for dishonesty binary and Table 2 shows the mean (standard deviation) scores for dishonesty magnitude across the three conditions.

**TABLE 1:** Study 1: Number of participants who provided at least one dishonest answer on the COVID‐21 vaccination registration form

	Answer Type	
Condition (Objective Risk)	All Honest	One or More Dishonest
Two times more deadly than all other COVID‐19 variants (*n* = 99)	71 (71.7%)	28 (28.3%)
10 times more deadly than all other COVID‐19 variants (*n* = 101)	68 (67.3%)	33 (32.7%)
100 times more deadly than all other COVID‐19 variants (*n* = 102)	65 (63.7%)	37 (36.3%)
Total (*N* = 302)	204 (67.5%)	98 (32.5%)

**TABLE 2:** Study 1: Mean number of dishonest answers to the 12 questions on the COVID‐21 vaccination registration form

Condition (objective risk)	Mean (standard deviation) number of dishonest answers per participant
Two times more deadly than all other COVID‐19 variants (*n* = 99)	0.6 (1.5)
10 times more deadly than all other COVID‐19 variants (*n* = 101)	0.6 (1.4)
100 times more deadly than all other COVID‐19 variants (*n* = 102)	1.1 (2.1)
Total (*N* = 302)	0.8 (1.7)

Scores for the single item that assessed participant's worry about the potential effects of COVID‐21 were multiplied by 10 to provide scores from 0 to 100. This item was then combined with the other two items used to measure perceived risk to form a scale labeled as ‘overall perceived risk’ (Cronbach's *α*  =  0.76). Table 3 provides means and standard deviations for the independent variables that we measured.

**TABLE 3:** Study 1: Means (standards deviations) for independent variables (*N* = 294–302)

Variable	Scale range	Mean (SD)
Overall perceived risk	0–100 (higher score = higher perceived risk)	65.7 (17.8)
Belief in a just world	1–6 (higher score = higher belief in just world)	3.7 (0.6)
Moral standards	1–5 (higher score = higher moral standards)	3.2 (0.4)
Risk‐taking propensity	0–10 (higher score = higher risk‐taking)	5.2 (1.9)
Political views	1–5 (1 is left‐wing; 5 is right‐wing)	2.5 (1.0)
Willingness to receive vaccinations	0–10 (higher score = higher willingness)	8.7 (1.6)

We first performed a logistic regression, with dishonesty binary as the outcome variable and both overall perceived risk and the condition (i.e., objective risk) as the predictor variables. For exploratory purposes, belief in a just world, moral standards, risk‐taking propensity, political views, vaccination willingness, age, gender, and education were also included as control variables. For the objective risk variable, the ‘two times more deadly’ condition was the baseline comparator, and for gender, ‘male’ was the baseline comparator.

The results (see Table 4) showed that only perceived risk and age had significant positive relationships with dishonesty binary. These findings did not provide support for Hypothesis 1, but did support Hypothesis 2. An exploratory *t*‐tests confirmed that perceived risk was significantly higher, *t*(300) = 3.29; *p* < 0.001, among participants who were dishonest (*M* = 70.5, *SD* = 18.2) compared to those who were honest (*M* = 63.4, *SD* = 17.2), and that age was significantly higher, *t*(300) = 3.68; *p* < 0.001, among participants who were dishonest (*M* = 34.5, *SD* = 8.6) compared to those who were honest (*M* = 30.8, *SD* = 8.2).

**TABLE 4:** Study 1: Logistic regression of assessed variables on dishonesty binary (N = 302)

Predictor variable	*b*	SE	*p*	Odds ratio (95% CI)
Overall perceived risk	0.022	0.008	0.006	1.02 (1.01, 1.04)
Condition (two times more deadly)	–	–	0.628	–
Condition (10 times more deadly)	0.204	0.324	0.528	1.23 (0.65, 2.31)
Condition (100 times more deadly)	0.312	0.329	0.343	1.37 (0.71, 2.60)
Belief in a just world	−0.190	0.214	0.375	0.83 (0.54, 1.26)
Moral standards	−0.093	0.381	0.807	0.91 (0.42, 1.92)
Risk‐taking propensity	0.044	0.073	0.546	1.05 (0.91, 1.20)
Political views	−0.121	0.141	0.389	0.89 (0.67, 1.17)
Willingness to receive vaccinations	0.007	0.087	0.931	1.01 (0.85, 1.19)
Age	0.054	0.16	<0.001	1.06 (1.02, 1.09)
Gender (male)	–	–	0.886	–
Gender (female)	0.217	0.441	0.623	1.24 (0.52–2.95)
Gender (other)	−20.133	28415.778	0.999	0.00
Education (bachelor's degree or above)	−0.086	0.272	0.753	0.92 (0.54, 1.57)
Constant	−3.231	1.830	0.077	0.04

A forced entry multiple linear regression was then performed to assess whether the dishonesty magnitude was related to objective and perceived risk. The outcome variable was dishonesty magnitude, and the predictor variables were objective risk and overall perceived risk. The same control variables were included for exploratory purposes. The correlations and coefficients are shown in Tables 5 and 6, respectively. The analysis identified overall perceived risk as the only significant predictors of dishonesty magnitude, with the model explaining around 9% of the variance. Again, these results supported Hypothesis 2, but not Hypothesis 1.

**TABLE 5:** Study 1: Correlations between assessed variables in the multiple regression (*n* = 294, using list‐wise deletion)

Variable	1.	2.	3.	4.	5.	6.	7.	8.	9.	10.	11.	12.	13.
1. Dishonesty magnitude	1												
2. Condition (two times more deadly)	−0.063	1											
3. Condition (10 times more deadly)	−0.060	−0.501***	1										
4. Condition (100 times more deadly)	0.124*	−0.501***	−0.497***	1									
5. Overall perceived risk	0.247***	−0.063	−0.003	0.066	1								
6. Belief in a just world	−0.055	−0.017	0.021	−0.004	−0.079	1							
7. Moral standards	0.036	−0.044	−0.026	0.071	0.053	0.019	1						
8. Risk‐taking propensity	0.048	0.033	0.053	‐.086	−0.012	0.025	−0.358***	1					
9. Political views	0.019	−0.020	−0.090	.110*	−0.015	0.119*	0.075	−0.030	1				
10. Willingness to receive vaccinations	0.083	−0.050	−0.038	.088	0.184***	0.000	0.036	−0.030	−0.057	1			
11. Age	0.098*	−0.034	0.020	.014	0.089	−0.006	0.180***	−0.064	0.151**	−0.075	1		
12. Gender	0.033	0.032	0.052	.084	0.083	0.011	0.147**	0.041	0.097*	0.027	0.094	1	
13. Education	−0.036	0.024	0.031	−0.056	0.102*	0.012	−0.083	−0.080	−0.208***	0.094	−0.027	0.020	1

*Note*. Each variable was scored so that a higher score corresponded to a higher magnitude of the construct (e.g., a higher score on the moral standards scale corresponded to a reportedly higher magnitude of moral standards). For political views, a higher (lower) score corresponded to a greater affiliation with right (left) wing political views.

* *p* < 0.05;.

** *p* < 0.01;.

*** *p* < 0.001.

**TABLE 6:** Study 1: Multiple regression of assessed variables on dishonesty magnitude (*n* = 294, using list‐wise deletion)

	Unstandardized coefficients		
Variable	*B*	*SE*	Standardized coefficients
Condition (two times more deadly)	0.038	0.226	0.011
Condition (100 times more deadly)	0.390	0.231	0.113
Overall perceived risk	0.020	0.005	0.226***
Belief in a just world	−0.096	0.151	−0.037
Moral standards	0.091	0.273	0.021
Risk‐taking propensity	0.060	0.052	0.072
Political views	−0.011	0.099	−0.007
Willingness to receive vaccinations	0.046	0.061	0.044
Age	0.015	0.011	0.079
Gender	0.084	0.295	0.017
Education	−0.158	0.193	−0.049
*R* ^2^			0.089
*F*(11, 280)			2.492**

*Note*. Variance inflation factor (VIF) and tolerance statistics showed no evidence of multicollinearity.

* *p* < 0.05;.

** *p* < 0.01;.

*** *p* < 0.001.

#### 2.1.4 Discussion

Our results revealed that lying was not related to objective risk, but was positively related to perceived risk. Broadly speaking, these findings are consistent with two key aspects of the extant literature. First, some studies indicate that individuals often neglect objective probabilities (e.g., numerical risk data) when making decisions, particularly when the focal outcome is mentally salient and affect‐rich (e.g., contracting a deadly disease) (Pachur et al., 2014; Rottenstreich and Hsee, 2001). Indeed, several studies suggest that objective probability data are often ineffective at raising perceived risks above a ‘threshold of concern’ (Knuth et al., 2014; Loewenstein et al., 2001; Robinson & Botzen, 2019) and it has been argued that in the context of the COVID‐19 pandemic the public have focused more on the potential adverse outcomes than on the probability data (DeSteno, 2020; see also Sieroń, 2020). Second, consistent with our findings, extant evidence indicate that risk perceptions are often strongly, and positively, related to the willingness to engage in self‐protective behaviors (e.g., Brewer et al., 2007; Dohle & Dawson, 2017; Floyd et al., 2000). What is somewhat novel about our findings is that they show risk perceptions may even motivate self‐protective behaviors (both in terms of being dishonest and in the extent of dishonesty) that are socially unacceptable or morally questionable, such as lying to obtain a vaccine.

In contrast to many past studies on age and dishonesty (see Jacobsen et al., 2018), we found that more older (cf. younger) participants were dishonest. We initially considered that our older participants could have perceived a greater health risk (Booth et al., 2021) and, therefore, were more motivated to behave dishonestly to increase their chances of getting the vaccination. We conducted an exploratory correlational analysis to test this possibility. While this identified a positive relationship between age and the perceived threat to life, it was just above the 0.05 significance threshold (*r* = 0.11, *N* = 292, *p* = 0.065). Hence, there may be other factors that influenced more of our older participants to be dishonest. For example, Jacobsen et al. (2018, p. 365) discuss the notion that dishonesty can be “mastered” with age and is typically rationalized using “self‐serving justifications” that protect the moral self. Therefore, in light of the greater threat to their health, our older participants may have skillfully weighed the benefits (high chance of obtaining a vaccine) and risks (low chance of being exposed as dishonest and not receiving the vaccine) of lying and concluded that dishonesty would provide a clear personal advantage that could be easily justified, thus protecting the moral self.

### 2.2 Study 2

Study 1 illustrated that the perceived risk of a pandemic disease may be closely related to the willingness to be dishonest in order to access risk management resources. We considered it possible that this relationship between perceived risk and dishonesty may not be unique to attempting to access a COVID vaccine. As mentioned earlier, several billion dollars were paid in the United Kingdom and the United States to fraudulent claimants during the early stages of the COVID‐19 lockdowns, indicating that dishonesty was extremely prevalent in financial contexts. However, the extent to which such dishonesty was related to perceived risk and whether the propensity to lie would vary according to whether an individual was trying to access health‐related or finance‐related resources was unclear. Hence, we conducted a second preregistered study (https://archive.org/details/osf‐registrations‐t9rga‐v1) that tested whether (a) there is a relationship between perceived financial risk during a pandemic and lying to obtain furlough payments, and (b) lying is greater among individuals trying to obtain vaccines or furlough payments. That is, Study 2 assessed Hypotheses 3 and 4.

#### 2.2.1 Participants

In November 2021, we recruited a sample of 204 adult prolific participants and paid each participant £1.50. The same selection criteria was employed as in Study 1, with the addition of requesting an equal gender balance and excluding participants from Study 1. No participants failed our IMC, but three participants were excluded for providing incomplete data. This left a final sample of 201 participants. The sample's mean age was 31.96 (*SD* = 8.5), 98 identified as female and 103 had obtained a bachelor's degree or higher.

#### 2.2.2 Materials and procedure

Via a Qualtrics survey, participants were randomly allocated to respond to either a health scenario or a financial scenario. The health scenario was identical to that used in Study 1, except that in all scenarios COVID‐21 was described as being “*10 times more deadly than all other known variants*.” In Study 2, participants in the health condition were asked to respond to three questions about their age and health status; one question was about age, one about experiencing flu‐like symptoms in the past 4 weeks, and a single question that asked if they had any of a list of 10 health conditions (these were the 10 health conditions that had been presented as 10 separate questions in Study 1). Three questions were used instead of 12 to create parity with the quantity of questions used in the financial condition (see below). All three questions could be answered “yes” or “no,” with positive responses being deemed dishonest. To measure perceived risk the same three questions were used from Study 1.

The financial scenario asked the participants to imagine that, “You are a small business owner in the UK, and this business provides the only source of income for you and your family. To save on costs, your business has always used a non‐UK bank account and is not part of the UK Government's Pay as You Earn (PAYE) scheme for paying employee's income tax (your employees are all paid in cash and they pay their own taxes).” As per Study 1, the scenario explained about the COVID‐21 pandemic, but then explained that a new lockdown would be imposed, which would likely lead to the liquidation of the participant's business. The scenario stated that financial support could be obtained for the business through the Government's new furlough scheme. However, participants were advised that there would only be enough funds in the furlough scheme to provide payments to one in every 10 businesses. Hence, businesses that wanted to be considered for the furlough support would need to complete the government's “COVID‐21 Furlough Payment Registration Form,” which asked participants three questions about their business. Participants were made aware that the government would prioritize giving the furlough payments to people who met any of the three criteria respectively set out in each of the three questions. The criteria were (i) the business must have created and started a ‘Pay as you earn’ (PAYE) payroll scheme on or before 30 October 2020 (ii) the business must have enrolled for PAYE online (iii) the business must have a UK bank account. As per Study 1, all three questions could be answers “yes” or “no,” with “yes” responses being deemed dishonest. All three criteria were closely based on the criteria used by the UK Government to determine eligibility for the Coronavirus Job Retention Scheme in 2021 (UK Government, 2021b).

To measure perceived risk in the financial condition, participants completed an 11‐point scale (0  =  not at all worried; 10  =  extremely worried) to indicate the extent of their worry about the potential effects of the lockdown on their business, and a 101‐point scale (0  =  impossible; 100  =  certain) to indicate the likelihood that the new lockdown would (i) have a negative impact on their business, and (iii) cause their business to collapse.

As in Study 1, participants completed the belief in a just world scale, the moral standards scale, the self‐reported risk‐taking question, and the political views question. Participants also used an 11‐point scale (0 = Not at all willing; 10 = Extremely willing) to report their general willingness to receive vaccines (health condition) or to receive financial benefits from the government (financial condition).

#### 2.2.3 Analysis and results

As per Study 1, we created two versions of the dependent variable for both conditions, with the dishonesty magnitude version ranging from zero to three. Tables 7 and 8 show, respectively, the counts for dishonesty binary and the mean (standard deviation) scores for dishonesty magnitude across both conditions.

**TABLE 7:** Study 2: Number of participants who provided at least one dishonest answer on the COVID‐21 vaccination registration form (health condition) or on the COVID‐21 furlough payment registration form (financial condition)

	Answer type	
Condition	All honest	One or more dishonest
Health (*n* = 101)	72 (71.3%)	29 (28.7%)
Financial (*n* = 100)	63 (63.0%)	37 (37.0%)
Total (*N* = 201)	135 (67.2%)	66 (32.8%)

**TABLE 8:** Study 2: Mean number of dishonest answers to the three questions on the COVID‐21 vaccination registration form (health condition) and on the COVID‐21 furlough payment registration form (financial condition)

Condition	Mean (standard deviation) number of dishonest answers per participant
Health (*n* = 100)	1.0 (1.3)
Financial (*n* = 101)	0.4 (0.7)
Total (*N* = 201)	0.7 (1.1)

As per the method used in Study 1, we formed a “perceived health risk” scale for the health condition (Cronbach's *α*  =  0.80) and a “perceived financial risk” scale for the financial condition (Cronbach's *α*  =  0.89). These scales were further merged to create a single “overall perceived risk” scale (*M* = 77.3, *SD* = 20.4). Table 9 provides means and standard deviations for the measures obtained across both conditions.

**TABLE 9:** Study 2: Means (standards deviations) for independent variables (*N* = 201)

Health condition (*n* = 101)		
Variable	Scale range	Mean (SD)
Perceived health risk	0–100 (higher score = higher perceived risk)	64.0 (18.5)
Belief in a just world	1–6 (higher score = higher belief in just world)	3.8 (0.6)
Moral standards	1–5 (higher score = higher moral standards)	3.2 (0.4)
Risk‐taking propensity	0–10 (higher score = higher risk‐taking)	5.4 (2.1)
Political views	1– 5 (1 is left‐wing; 5 is right‐wing)	2.5 (1.1)
Willingness to receive vaccinations	0–10 (higher score = higher willingness)	8.3 (1.8)

Financial condition (*n* = 100)		
Variable	Scale range	Mean (SD)
Perceived financial risk	0–100 (higher score = higher perceived risk)	90.8 (11.6)
Belief in a just world	1–6 (higher score = higher belief in just world)	3.7 (0.6)
Moral standards	1–5 (higher score = higher moral standards)	3.2 (0.4)
Risk‐taking propensity	0–10 (higher score = higher risk‐taking)	5.5 (2.1)
Political views	1–5 (1 is left‐wing; 5 is right‐wing)	2.4 (0.9)
Willingness to receive financial benefits	0–10 (higher score = higher willingness)	8.3 (1.6)

To test Hypothesis 3, we first performed a Pearson correlation test to examine the relationship between dishonesty magnitude (using only the data from the financial condition) and perceived financial risk. This identified no significant relationship, *r* = 0.04, *N* = 100, *p* = 0.677, between the two variables. We then performed a *t*‐test to examine whether perceived financial risk varied significantly between participants who were either honest or dishonest in the financial condition. Again, no significant difference, *t*(98) = 0.86, *N* = 100, *p* = 0.390, in perceived financial risk was found between participants who were honest (*n *= 63, *M* = 91.5, *SD* = 9.7) and those who were dishonest (*n* = 37, *M* = 89.5, *SD* = 14.4). Hence, Hypothesis 3 was not supported. For exploratory purposes, we repeated these two tests using data from the health condition only. Consistent with the results of Study 1, the Pearson correlation test found a significant positive relationship, *r* = 0.28, *N* = 101, *p* = 0.005, between dishonesty magnitude and perceived health risk. Relatedly, a *t*‐test found that perceived health risk was significantly higher, *t*(99) = 2.64, *N* = 101, *p* = 0.010, among dishonest participants (*n* = 29, *M* = 71.5, *SD* = 18.0) than among honest participants (*n* = 72, *M* = 61.0, *SD* = 17.9).

To address Hypothesis 4, we first performed a logistic regression, with dishonesty binary as the outcome variable and the condition (health vs. financial) as the predictor variable. For exploratory purposes, overall perceived risk, belief in a just world, moral standards, risk‐taking propensity, political views, age, gender, and education were also included in the regression. The results (see Table 10) showed that only age had significant positive relationship with dishonesty binary. Exploratory *t*‐tests showed that, in the health condition, age was significantly higher, *t*(37.917) = 3.07; *p* = 0.004, among participants who were dishonest (*M* = 40.2, *SD* = 14.6) compared to those who were honest (*M* = 31.2, *SD* = 9.5). However, in the financial condition, there was no significant difference, *t*(98) = 0.101; *p* = 0.920, among participants who were dishonest (*M* = 32.9, *SD* = 10.1) compared to those who were honest (*M* = 32.7, *SD* = 9.6).

**TABLE 10:** Study 2: Logistic regression of assessed variables on dishonesty binary (N = 201)

Predictor Variable	*b*	SE	*p*	Odds ratio (95% CI)
Condition	0.128	0.432	0.766	1.14 (0.49, 2.65)
Overall perceived risk	0.021	0.012	0.064	1.02 (1.00, 1.04)
Belief in a just world	0.248	0.285	0.384	1.28 (0.73, 2.24)
Moral standards	−0.753	0.417	0.071	0.47 (0.21, 1.07)
Risk‐taking propensity	−0.106	0.084	0.207	0.90 (0.76, 1.07)
Political views	0.120	0.168	0.475	1.13 (0.81, 1.57)
Age	0.033	0.15	0.025	1.03 (1.00, 1.06)
Gender (male)	–	–	0.828	–
Gender (female)	−0.204	0.332	0.538	0.82 (0.43, 1.56)
Gender (other)	−20.593	28,325.128	0.999	0.000
Education (bachelor's degree or above)	−0.102	0.324	0.752	0.90 (0.48, 1.70)
Constant	−1.666	1.988	0.402	0.19

We then performed a forced entry multiple linear regression to assess whether the *extent of* dishonesty was significantly greater in the financial (cf. health) condition. The outcome variable was dishonesty magnitude and the predictor variable was condition. As in the logistic regression, the same predictors were included for exploratory purposes. The correlations and coefficients are shown in Tables 11 and 12, respectively. The analysis identified age as the only significant predictor of dishonesty magnitude, with the model explaining around 9% of the variance. However, an exploratory Pearson correlation test showed that the relationship between age and dishonesty magnitude fell just short of being significant, *r* = 0.14, *N* = 210, *p* = 0.054. In conclusion, the linear regression did not find dishonesty to be greater among individuals trying to access furlough payments (cf. vaccines) and, therefore, Hypothesis 4 was also not supported.

**TABLE 11:** Study 2: Correlations between assessed variables in the multiple regression (*N* = 201, using list‐wise deletion)

Variable	1.	2.	3.	4.	5.	6.	7.	8.	9.	10.
1. Dishonesty magnitude	1									
2. Condition	−0.263***	1								
3. Overall perceived risk	0.269***	−0.657***	1							
4. Belief in a just world	0.064	0.042	−0.097	1						
5. Moral standards	−0.106	0.072	0.003	0.137*	1					
6. Risk‐taking propensity	−0.010	−0.034	−0.039	0.063	−0.341***	1				
7. Political views	0.14	0.047	−0.056	0.266***	0.114	0.141*	1			
8. Age	0.136*	0.046	0.012	0.013	0.022	−0.050	0.149*	1		
9. Gender	−0.026	−0.062	0.181**	0.001	0.026	−0.218***	−0.082	−0.092	1	
10. Education	−0.015	0.124*	−0.025	0.047	0.026	−0.153*	−0.090	−0.018	−0.063	1

*Note*. Each variable was scored so that a higher score corresponded to a higher magnitude of the construct (e.g., a higher score on the moral standards scale corresponded to a reportedly higher magnitude of moral standards). For political views, a higher (lower) score corresponded to a greater affiliation with right (left) wing political views. For condition, financial was coded as “0” and health was coded as “1.” For gender, male was coded as “1” and female was coded as “2.”

* *p* < 0.05;.

** *p* < 0.01;.

*** *p* < 0.001.

**TABLE 12:** Study 2: Multiple regression of assessed variables on dishonesty magnitude (*N* = 201, using list‐wise deletion)

	Unstandardized coefficients		
Variable	*B*	*SE*	Standardized coefficients
Condition	−0.329	0.204	−0.148
Overall perceived risk	0.010	0.005	0.181
Belief in a just world	0.197	0.134	0.105
Moral standards	−0.318	0.190	−0.124
Risk‐taking propensity	−0.017	0.040	−0.032
Political views	0.000	0.080	0.000
Age	0.014	0.007	0.140*
Gender	−0.015	0.153	−0.007
Education	0.007	0.155	0.003
*R* ^2^			0.085
*F*(9, 191)			3.065**

*Note*. Variance inflation factor (VIF) and tolerance statistics showed no evidence of multicollinearity.

* *p* < 0.05.

** *p* < 0.01.

*** *p* < 0.001.

#### 2.2.4 Discussion

Study 2 found that, in the financial condition, the participant's perception of the risk to their finances was not related to their willingness to lie in order to increase the chance of obtaining furlough payments. This finding is in contrast to the extant literature that has reported a strong positive relationship with perceived risk and risk management behaviors. To some extent, it is also in contrast to the results obtained in the vaccine scenarios in Studies 1 and 2, which showed that the participant's risk perceptions were significantly related to their dishonesty. This indicates that the influence of perceived risk on dishonesty is not uniform across all contexts. Indeed, taken together, these findings indicate that individuals probably place a much higher value on their personal health (cf. financial income) and, therefore, are more willing to take greater risks (e.g., prosecuted for criminal behavior) or to deviate from moral standards by lying in order to protect their health. This finding points toward the possibility that during the COVID‐19 pandemic the high levels of financial fraud observed in countries such as the United Kingdom and United States may have been driven more by nefarious motives and/or perceived personal benefits (e.g., greed, selfishness) than by worries about suffering financial hardship. Relatedly, past studies on general benefit fraud have often found that fraud is motivated more by greed and opportunity than by financial needs (Hessing et al., 1993; Tunley, 2011).

While approximately one‐third (i.e., 32.8%) of all participants in Study 2 were dishonest, we found no significant difference between dishonesty (both in its binary form and in terms of its magnitude) in the furlough and vaccine scenarios. This finding was in contrast to Hypothesis 4, which predicted that dishonesty would be greater in the furlough scenario. We considered two plausible explanations for this finding. First, Hypothesis 4 was largely based on the vast and well‐documented magnitude of the financial fraud that occurred in multiple countries during the COVID‐19 pandemic. However, it is plausible that the level of vaccine fraud was just as high during the pandemic, but that this was not well documented or reported. Hence, our results may simply reflect a reality in which dishonesty may have been equally prevalent among those attempting to obtain vaccines and those attempting to obtain furlough payments. Second, our finding that dishonesty did not differ significantly between the two conditions might indicate that the external context (i.e., vaccine vs. furlough scenarios) was less relevant to the decision to behave dishonestly, and that the decision may have been governed more by an intrinsic trait that one might expect to be equally evident in any randomly selected sample of a population. Indeed, there is an emerging literature that suggests (dis)honesty is a personality trait and that one might expect to find this trait to greater or lesser extents across all populations (see Fleeson et al., 2022; Johnson et al., 2011). Hence, the lack of support for Hypothesis 4 may have also been a reflection of a resilience in “trait dishonesty” to the contextual variations in our two scenarios.

It is also worth noting that it was only in the vaccine scenario that we observed greater dishonesty among the older (cf. younger) participants. As discussed previously, this may have been because the importance of personal health provided the more vulnerable older participants with a stronger self‐serving justification for being dishonest. By contrast, it seems unlikely that older individuals would be more vulnerable to the financial pressures described in our furlough scenario.

### 2.3 Study 3

As discussed earlier, the high levels of fraudulent claims for financial support during the COVID‐19 pandemic suggested that many people interpreted the probability of detection as being negligible. However, to the best of our knowledge, there had been no studies that had assessed the role that the probability of detection might play in deterring dishonesty during a pandemic. As highlighted by our first study 1, the relatonships between dishonesty and both perceived risk and objective risk can differ. Hence, we considerd it plausible that while the perceived risk and the objective risk of dishonesty being detected could both influence dishonesty, this influence might vary in magnitude. To examine this possibility and, therefore, to address Hypotheses 5 and 6 we conducted Study 3 (OSF preregistration: https://archive.org/details/osf‐registrations‐jmq8g‐v1). In light of the large quantities of money that were obtained fraudulently during the COVID‐19 pandemic, we elected to focus only on lying to access furlough payments when testing these two hypotheses.

#### 2.3.1 Participants

In December 2021, a sample of 270 adult Prolific participants was recruited and paid £1.00 each. The same selection criteria was employed as in Study 2, with the addition of excluding participants from Study 2. No participants failed our IMC. The mean age was 40.4 (*SD* = 13.9), 135 identified as female and 152 had obtained a bachelor's degree or higher.

#### 2.3.2 Materials and procedure

Via Qualtrics, participants responded to the financial scenario used in Study 2. However, using a between‐subjects design, the scenario described the probability of dishonesty detection as being either zero, 1 in 100, or 1 in 10. In the “zero condition,” the manipulated text read, “*… the government has announcement that it does not have the capacity or resources to verify whether people have provided honest answers on the furlough payments registration form*.” In the other two conditions, the text was varied: “*… the government has announcement that it will check one in every 100 [one in every 10] furlough payment registration forms … a government spokesperson has stated that they will start a criminal prosecution against anyone who is found to have provided inaccurate information …*” Participants were randomly allocated to one of the three conditions and, as per Study 2, after reading the scenario participants completed the three questions on the *COVID‐21 Furlough Payment Registration Form*.

We used the same three questions from Study 2 to assess the participant's perceived risk of the lockdowns. To assess the perceived risk of detection, participants were asked to use an 11‐point scale (0  =  not at all worried; 10  =  extremely worried) to indicate the extent to which they would be worried about being prosecuted if they provided inaccurate information on the furlough payment registration form.

#### 2.3.3 Analysis and results

First, we created a dishonesty binary and a dishonesty magnitude dependent variable. Table 13 shows the counts for dishonesty binary and Table 14 shows the mean (standard deviation) scores for dishonesty magnitude across the three conditions. Following the approach used in Study 2, an overall perceived financial risk scale was created (Cronbach's *α*  =  0.86). Table 15 provides means and standard deviations for the measures obtained.

**TABLE 13:** Study 3: Number of participants who provided at least one dishonest answer on the COVID‐21 furlough payment registration form

	Answer type	
Condition (objective probability of detection)	All honest	One or more dishonest
Zero (*n* = 90)	54 (60.0%)	36 (40.0%)
1 in 100 (*n* = 89)	63 (70.8%)	26 (29.2%)
1 in10 (*n* = 91)	74 (81.3%)	17 (18.7%)
Total (*N* = 270)	191 (70.7%)	79 (29.3%)

**TABLE 14:** Study 3: Mean number of dishonest answers to the three questions on the COVID‐21 furlough payment registration form

Condition (objective probability of detection)	Mean (standard deviation) number of dishonest answers per participant
Zero (*n* = 90)	1.0 (1.4)
1 in 100 (*n* = 89)	0.7 (1.2)
1 in 10 (*n* = 91)	0.5 (1.0)
Total (*N* = 270)	0.7 (1.2)

**TABLE 15:** Study 3: Means (standards deviations) for independent variables (*N* = 270)

Variable	Scale range	Mean (SD)
Overall perceived risk to business	0–100 (higher score = higher perceived risk)	88.6 (14.4)
Perceived risk of prosecution	0–10 (higher score = higher perceived risk)	8.1 (2.3)
Belief in a just world	1–6 (higher score = higher belief in just world)	3.8 (0.7)
Risk‐taking propensity	0–10 (higher score = higher risk‐taking)	4.6 (2.3)
Political views	1–5 (1 is left‐wing; 5 is right‐wing)	2.6 (0.9)
Willingness to receive financial benefits	0–10 (higher score = higher willingness)	7.5 (2.2)

To address Hypothesis 5, we first performed a 2 × 3 cross‐tabulated chi‐square test, treating dishonesty binary (honest, dishonest) as the dependent variable and probability of detection (zero, 1 in 100, 1 in 10) as the independent variable. This identified a significant difference between the observed and expected counts, *X^2^* = 9.94, *df* = 2, *p* = 0.007, with the number of dishonest participants declining as the probability of detection increased across the three conditions (see Table 13). We then performed a one‐way ANOVA, with dishonesty magnitude as the dependent variable and probability of detection as the independent variable. This showed that dishonesty magnitude significantly declined, *F*(2,267) = 5.08, *p* = 0.007, as the probability of detection increased across the three conditions (see Table 14). Bonferroni *post hoc* tests identified a significant difference in dishonesty magnitude between the zero and the 1 in 10 probability of detection conditions, *p *= 0.005, but not between any other pairs of conditions, *p*s ≥ 0.283. These results supported Hypothesis 5.

To test Hypothesis 6, we performed a Pearson correlation test to examine the relationship between perceived risk of detection and dishonesty magnitude. Consistent with Hypothesis 6, this identified a significant negative relationship, *r* = −0.17, *N* = 270, *p* = 0.005. We then performed a *t*‐test to examine whether perceived risk of detection varied significantly between the honest and dishonest participants. This identified that the perceived risk of detection was significantly greater, *t*(268) = 2.79, *N* = 270, *p* = 0.006, among honest (*n *= 191, *M* = 8.3, *SD* = 2.3) than among dishonest (*n* = 79, *M* = 7.4, *SD* = 2.2) participants. Hence, Hypothesis 6 was supported.

#### 2.3.4 Discussion

Study 3 revealed that both the objective probability and perceived risk of dishonesty being detected have a negative relationship with lying *and* the extent of lying. This suggest that in the COVID‐19 context the objective probability and perceived risk of being detected may have both been too low to deter such behavior. Therefore, we recommend that in future pandemics (and other crises), greater efforts be made to explicitly communicate the probability of detection. Moreover, as demonstrated by our findings, the probability of detection may need to be sufficiently high (e.g., 1 in 10 or greater) to have a significant impact on reducing fraudulent claims. Indeed, during the actual COVID‐19 pandemic, the probability of detection may have been perceived (rightly or wrongly) as being much lower than 1 in 10 and, consequently, many individuals may not have been deterred from making fraudulent claims.

## 3 GENERAL DISCUSSION

Across three studies, we examined the relationship between risk and dishonesty in a global pandemic. Study 1 showed that heightened perceptions of the health risks posed by a pandemic were positively related with lying in order to protect one's health. While Study 2 also found evidence of the same relationship, the study revealed that no such association was evident between financial risk perceptions and lying to protect one's financial position. Study 3 showed that both the objective probability and perceived risk of dishonest being detected had a negative relationship with lying. Across these studies, two other notably findings were that lying was (i) associated with older age in a health‐related context and (ii) consistently evident in approximately one‐third of all of our samples (32.5% in Study 1, 32.8% in Study 2, 29.3% in Study 3).

Our studies offer two additional and important contributions. First, we found that lying positively correlates with health risk perceptions, but not with personal moral standards. That is, (dis)honesty during a pandemic appears much more heavily determined by concerns about adverse health outcomes than it does by an intrinsic sense of right and wrong. Arguably, this corresponds with a key tenet of prospect theory, which purports that individuals become risk seeking when faced with potential losses (Kahneman and Tversky, 1979). Our results suggest that when faced with a potential loss of great magnitude (e.g., contracting a deadly disease), the extent to which individuals are motivated to take risks can include engaging in immoral behaviors. Relatedly, this finding also corresponds with a key strand of the theory of Bounded Ethicality, which argues that individuals often have to make difficult trade‐offs between scared values (Bazerman, Curhan, Moore & Valley, 2000; Chugh, Bazerman & Banaji, 2005). In our first two studies, it appears that many participants elected to trade the virtuous value of honesty in favor of the fundamental value of self‐preservation. As discussed previously, economic theories have long asserted that individuals weigh the costs and benefits of acting dishonestly and, thereafter, engage in the course of action that maximizes self‐interest (Allingham & Sandmo, 1972; Becker, 1968). As suggested by our findings, for many individuals the existential threat of a deadly disease may prove to be a powerful driver of acting dishonestly to maximize self‐interest.

Second, our studies identify some important nuances concerning the relationship between risk perceptions and risk‐related behaviors. As identified in Studies 1 and 2 (see also Floyd et al., 2000), there is a strong positive relationship between health risk perceptions and engagement in health protective behaviors. Notably, Studies 1 and 2 showed that this relationship could even extend to engagement in health protective behaviors that involve dishonesty. However, Study 2 highlighted that this relationship was not evident in the financial context. Therefore, other motivational forces (e.g., greed, opportunism, perceived benefits) might prompt dishonesty in financial contexts during a pandemic. Gummerum et al. (2014) also found that the positive relationship between heightened risk perceptions and risk‐related behaviors was only evident in the health domain. Hence, it should not be assumed that risk perceptions are inextricably linked to protective behaviors in all domains. Moreover, Study 3 did show that, in a financial context, the perceived risk of dishonesty being detected was related to a reduction in dishonest behavior. Hence, it appears that the perceived risk of financial hardship may not always motivate engagement in dishonest behaviors, but the perceived risk of punishment may deter such behavior.

### 3.1 Considerations for policy, risk communication, and theory

Dishonest behaviors such as vaccine fraud and financial fraud have created substantial problems during the COVID‐19 pandemic. Not only have these behaviors led to the loss of billions of dollars but, more importantly, they may have prevented some of society's most vulnerable people from accessing vital resources (e.g., vaccines). Therefore, in future pandemics, governments should aim to adopt policies and practices that help to reduce such behaviors. Our third study provides evidence that dishonesty can be reduced by explicitly communicating the probability of detection and by making that probability sufficiently high. Nonetheless, governments should be mindful that it can be challenging to communicate probabilistic data in a way that is easily comprehended by lay audiences. Fortunately, there is a wealth of empirical evidence that policy makers could draw upon for guidance on communicating numerical risk information (see Lipkus, 2007; Visschers et al., 2009).

During a pandemic, public risk perceptions can be central to motivating relevant health protective behaviors. However, as our studies demonstrate, heightened risk perceptions may also be associated with dishonest behavior. This suggest that policy makers should aim to strike a careful balance between raising public awareness of the pandemic disease sufficient to motivate protective behaviors, but not raising fear levels to such heights that individuals become motivated to take unethical measures to protect themselves. Again, we recommend that, to achieve this balance, policy makers draw upon the range of extent evidence for guidance on communicating health risk magnitudes (see Gigerenzer & Edwards, 2003; Fischhoff et al., 2000; Ropeik & Slovic, 2003). Furthermore, in terms of detecting dishonesty, we recommend that governments focus their resources on those individuals and groups whom, according to research evidence, may have a greater propensity for dishonesty. Our studies suggest that this propensity may be greatest in a pandemic among older individuals and those who have heightened health risk perceptions.

As alluded to in our previous discussions, there is the potential to utilize our findings to develop and test a theoretical model concerning the general role of perceived risk in dishonest behaviors. Specifically, such a model might predict that dishonest behaviors are positively associated with perceived risk, but only when the level of risk is perceived to exceed an intrinsic threshold for virtuous conduct (i.e., the point beyond which the individual concludes that dishonesty can be justified on the basis that selfish interests should be prioritized over adherence to personal and/or socially accepted moral standards). The model could include trait honesty and the probability of dishonesty detection as moderating factors. Empirical examinations of such a model could provide novel and important insights into a range of nefarious and harmful human behaviors and, therefore, could also illuminate potential approaches for strategies and interventions that aim to minimize dishonest conduct.

### 3.2 Limitations and future directions

A key contribution from our studies is the identification of the key role played by the perceived risk of a pandemic disease in providing a subjective rationalization for acting dishonestly to obtain a vaccine. When considering the generalizability of this finding, it is important to note that our participants were individuals who had already obtained COVID‐19 vaccines and, therefore, our findings may not representative of the behaviors of those individuals who had previously declined such vaccines. Indeed, research on the willingness of individuals to obtain vaccines, both before and during the COVID‐19 pandemic, has highlighted the problem of “vaccine hesitancy” (i.e., a reluctance to obtain a vaccine due to safety and efficacy concerns: Freimuth et al., 2017; Larson et al., 2022; WHO, 2019). In a general population, one might expect to find lower levels of willingness to obtain a vaccine and, therefore, to find fewer instance of lying to obtain a vaccine. Nonetheless, our findings do highlight that, in contrast to vaccine hesitancy, there can also be a level of “vaccine urgency” that is so great that individuals will engage in questionable actions to obtain the vaccine. In other words, our results suggest that the influence of heightened perceived risks on decision behaviors may be so powerful that it motivates behaviors that are largely in contrast with both prevailing social trends and socially expected standards of behavior. Hence, we believe that there could be much value in future research that examines the more general role of heightened levels of perceived risk in motivating a range of socially undesirable behaviors. For example, it would be beneficial to understand the extent to which certain dishonest behaviors (e.g., crimes, moral indiscretions, cheating) are a function of a desire to protect oneself or significant others from specific perceived risks (e.g., financial hardships, illness, social judgments). Similarly, it would be helpful to understand if risk perceptions and behaviors are associated with an intrinsic propensity toward dishonesty. Indeed, research has already shown that less honest individuals (measured as a personality trait) perceive a lower risk of driving while using a mobile phone and more frequently drive while on a mobile phone (Sween et al., 2017). More research on these topics would provide valuable insights concerning the risk‐related motivational forces that underlie nefarious and harmful behaviors and, therefore, could illuminate potential avenues for interventions that address these forces rather than the symptomatic behaviors.

There are some further limitations to our studies that should also be considerd. First, our studies did not identify what factors primarily motivate dishonesty in financial contexts during a pandemic. For example, it would be useful to know if the motivation to act dishonestly is affected by whether the individual believes that they or someone else with benefit from such behavior. Research exploring these topics could prove valuable, particularly in helping governments to develop empirically informed prevention strategies. Second, our research used hypothetical scenarios and, therefore, elicited decisions of a hypothetical nature. While there is a wealth of evidence that shows hypothetical scenarios often obtain data with high external validity (see Aguinis & Bradley, 2014), future studies should gather data from environments that are more naturalistic. In a real‐world context, one might expect that the more impactful prospect of dishonesty being detected and punished might result in fewer instances of dishonesty. However, as illustrated by our third study and several past studies, we would argue that the prevalence of dishonesty is still likely to be a function of the probability of detection. Future studies could build on the findings from our third study by examining whether the extent to which the probability of detection influences dishonesty varies between different contexts during a pandemic (e.g., health, financial, social, etc.). Third, we recruited our participants from prolific.ac and, therefore, it could be argued that these individuals may behave differently to other samples. While it is possible that samples recruited from prolific.ac may feature a greater prevalence of some characteristics (e.g., technologically active), research evidence does show that prolific.ac samples typically provide high quality, reliable data, and do not demonstrate a disproportionate level of dishonesty compared to participants recruited using other methods (Palan & Schitter, 2018; Peer et al., 2017). Fourth, it should be noted that in Study 1, we inadvertently recruited a sample with a gender imbalance. However, we found no evidence to suggest dishonesty was related to gender in any of our three studies. Finally, a growing body of work has identified the influential role of social factors, such as norms and values, in driving and deterring dishonesty (see Gino et al., 2009; Pierce & Balasubramanian, 2015). Hence, future research should examine the role that social factors and “behavioral nudges” could play in eliciting or discouraging dishonest behaviors in contexts where there is a high level of risk.

## 4 CONCLUSION

Numerous factors can influence dishonest behaviors. In our three studies, we focused on the influential role played by risk in the context of a global pandemic. We found that health risk perceptions, but not financial risk perceptions, were positively related to dishonesty. We also found that the perceived risk of dishonesty being detected was negatively related to dishonest behavior. These results suggest that health risk perceptions play a central role in extent to which individuals engage in dishonest behaviors during a pandemic, and may be indicative of the wider role that risk perceptions more generally play in immoral or illegal behaviors. Implementing empirically‐informed approaches that can help to reduce and detect dishonesty could prove to be of immense value in future crises. Our results illuminate some potential ways in which such approaches could be designed.

## Supporting information

SUPPLEMENTARY MATERIALS
