# Supplementary material for: The role of perceived risk on dishonest decision making during a pandemic
Source: Risk Anal. 2022 Dec 12;44(12):2762–79. doi: 10.1111/risa.14082 (PMC11669558; doi:10.1111/risa.14082)
Supplement: Supplementary file 1 — SUPPLEMENTARY MATERIALS [file RISA-44-2762-s001.docx]

**The Role of Perceived Risk on Dishonest Decision Making During a Pandemic**

**SUPPLEMENTARY MATERIALS**

**VACCINATION SCENARIO**

Imagine the following scenario. You are 40 years old, you have no known designated health conditions, and you have not experienced any flu-like symptoms in the past year.

A few days ago, scientists identified a new variant of COVID-19 that is two times (10 times/100 times) more deadly than all other known variants of the disease. In addition, none of the existing COVID-19 vaccines offer any form of protection against this new variant. The new variant has been named COVID-21.

Fortunately, scientists have confirmed that within the next two weeks they can produce a vaccine that will offer 95% protection against COVID-21. The only problem is that the vaccine manufacturers can only produce 5,000,000 doses of the new vaccine for people in your country of residence. Because your country of residence has a population of 50,000,000 people, this means that only one in every 10 people will be able to receive the COVID-21 vaccine. It is unknown how much longer you will have to wait for the next round of vaccinations, but it will be at least twelve months if not more.

The government have decided that anyone who wishes to receive the COVID-21 vaccine must first submit a registration form online. The government has stated that they will prioritise giving the vaccine to people who meet any of the following criteria:

- You are aged 60 years or over
- You have experienced flu-like symptoms in the past four weeks
- You have a designated health condition that makes you extremely vulnerable to getting seriously ill from COVID-21. The designated health conditions are:
  - long-term lung conditions (such as severe asthma, bronchiectasis and cystic fibrosis)
  - long-term conditions affecting the heart or blood vessels (such as congenital heart disease, heart failure and peripheral arterial disease)
  - diabetes
  - chronic kidney disease
  - long-term liver conditions (such as cirrhosis and hepatitis)
  - conditions affecting the brain or nerves (such as dementia, Parkinson's disease, motor neurone disease, multiple sclerosis, epilepsy, cerebral palsy or stroke)
  - learning disabilities
  - a condition or treatment that makes you more likely to get infections (such as HIV or some treatments for lupus, psoriasis or rheumatoid arthritis)
  - severe mental conditions (such as schizophrenia and bipolar disorder)
  - severe obesity (a BMI of 40 or above)

Due to the need to vaccinate people as soon as possible, the government has announcement that it does not have the capacity or resources to verify whether people have provided honest answers on the COVID-21 vaccine registration form. Indeed, a government spokesperson has stated that they will not have enough time and resources to verify people’s answers on the form before or after the vaccines are administered.

You start to think about how the new COVID-21 virus is two times (10 times/100 times) more deadly than all other variants of COVID-19 and you contemplate what could happen if you became infected with it. You decide to complete the COVID-21 vaccine registration form. With the above scenario in mind, please now complete the following section of the COVID-21 vaccine registration form:

**COVID-21 Vaccine Registration Form**

Please indicate (‘yes’ or ‘no’) whether you meet any of the following criteria:

- You are aged 60 years or over
- You have experienced flu-like symptoms in the past four weeks
- You have a designated health condition that makes you extremely vulnerable to getting seriously ill from COVID-21. The designated health conditions are:
  - long-term lung conditions (such as severe asthma, bronchiectasis and cystic fibrosis)
  - long-term conditions affecting the heart or blood vessels (such as congenital heart disease, heart failure and peripheral arterial disease)
  - diabetes
  - chronic kidney disease
  - long-term liver conditions (such as cirrhosis and hepatitis)
  - conditions affecting the brain or nerves (such as dementia, Parkinson's disease, motor neurone disease, multiple sclerosis, epilepsy, cerebral palsy or stroke)
  - learning disabilities
  - a condition or treatment that makes you more likely to get infections (such as HIV or some treatments for lupus, psoriasis or rheumatoid arthritis)
  - severe mental conditions (such as schizophrenia and bipolar disorder)
  - severe obesity (a BMI of 40 or above)

**END OF REGISTRATION FORM**

*********

**FURLOUGH SCENARIO**

Imagine the following scenario. You are a small business owner in the UK, and this business provides the only source of income for you and your family. To save on costs, your business has always used a non-UK bank account and is not part of the UK Government’s Pay as You Earn (PAYE) scheme for paying employee’s income tax (your employees are all paid in cash and they pay their own taxes).

A few days ago, scientists in the UK identified a new variant of COVID-19 that is 10 times more deadly than all other known variants of the disease. In addition, none of the existing COVID-19 vaccines offer any form of protection against this new variant. The new variant has been named COVID-21.

Due to the severity of COVID-21, the Government has decided to impose another lockdown for at least three months. Unfortunately, the new lockdown will impose further financial hardship on your business. So far, you have just about managed to keep your business afloat, but any more hardship is likely to lead to the liquidation your business. Luckily, the government is offering a new and more generous furlough scheme that will pay your employees’ wages and provide financial support for your business. The only problem is that the government can only afford to offer the furlough payments to 50,000 businesses. Because in the UK there are 500,000 businesses, this means that only one in every 10 businesses will be able to receive the furlough payments.

The government have decided that any business owner who wishes to receive the furlough payments must first submit a registration form online. The government has stated that they will prioritise giving the furlough payments to businesses that meet any of the following criteria:

- Your business must have created and started a ‘Pay as you earn’ (PAYE) payroll scheme on or before 30 October 2020
- Your business must have enrolled for PAYE online
- Your business must have a UK bank account

Due to the need to allocate the furlough payments as soon as possible, the government has announcement that it does not have the capacity or resources to verify whether people have provided honest answers on the furlough payments registration form. Indeed, a government spokesperson has stated that they will not have enough time and resources to verify people’s answers on the form before or after the furlough payments are administered.

You start to think about how the lockdown will affect your business and the severe financial implications it might have for your business and personal finances. You decide to complete the COVID-21 furlough payments registration form. With the above scenario in mind, please now complete the COVID-21 furlough payments registration form:

**COVID-21 Furlough Payment Registration Form**

Please indicate (‘yes’ or ‘no’) whether your business meets any of the following criteria:

- Your business has created and started a ‘Pay as you earn’ (PAYE) payroll scheme on or before 30 October 2020
- Your business has enrolled for PAYE online
- Your business has a UK bank account

**END OF REGISTRATION FORM**

*****
